# Microplastic effects on carbon cycling processes in soils

**DOI:** 10.1371/journal.pbio.3001130

**Published:** 2021-03-30

**Authors:** Matthias C. Rillig, Eva Leifheit, Johannes Lehmann

**Affiliations:** 1 Freie Universität Berlin, Institute of Biology, Berlin, Germany; 2 Berlin-Brandenburg Institute of Advanced Biodiversity Research (BBIB), Berlin, Germany; 3 Soil and Crop Sciences, College of Agriculture and Life Sciences, Cornell University, Ithaca, New York, United States of America

## Abstract

Microplastics (MPs), plastic particles <5 mm, are found in environments, including terrestrial ecosystems, planetwide. Most research so far has focused on ecotoxicology, examining effects on performance of soil biota in controlled settings. As research pivots to a more ecosystem and global change perspective, questions about soil-borne biogeochemical cycles become important. MPs can affect the carbon cycle in numerous ways, for example, by being carbon themselves and by influencing soil microbial processes, plant growth, or litter decomposition. Great uncertainty surrounds nano-sized plastic particles, an expected by-product of further fragmentation of MPs. A major concerted effort is required to understand the pervasive effects of MPs on the functioning of soils and terrestrial ecosystems; importantly, such research needs to capture the immense diversity of these particles in terms of chemistry, aging, size, and shape.

## Abstract 2

This Essay proposes that a major concerted effort is required to understand the pervasive effects of microplastics on the functioning of soils and terrestrial ecosystems, including the immense diversity of microplastic particles in terms of their chemistry, aging, size, and shape.

## Introduction: Microplastic as a factor of global change

**Fig 1:**
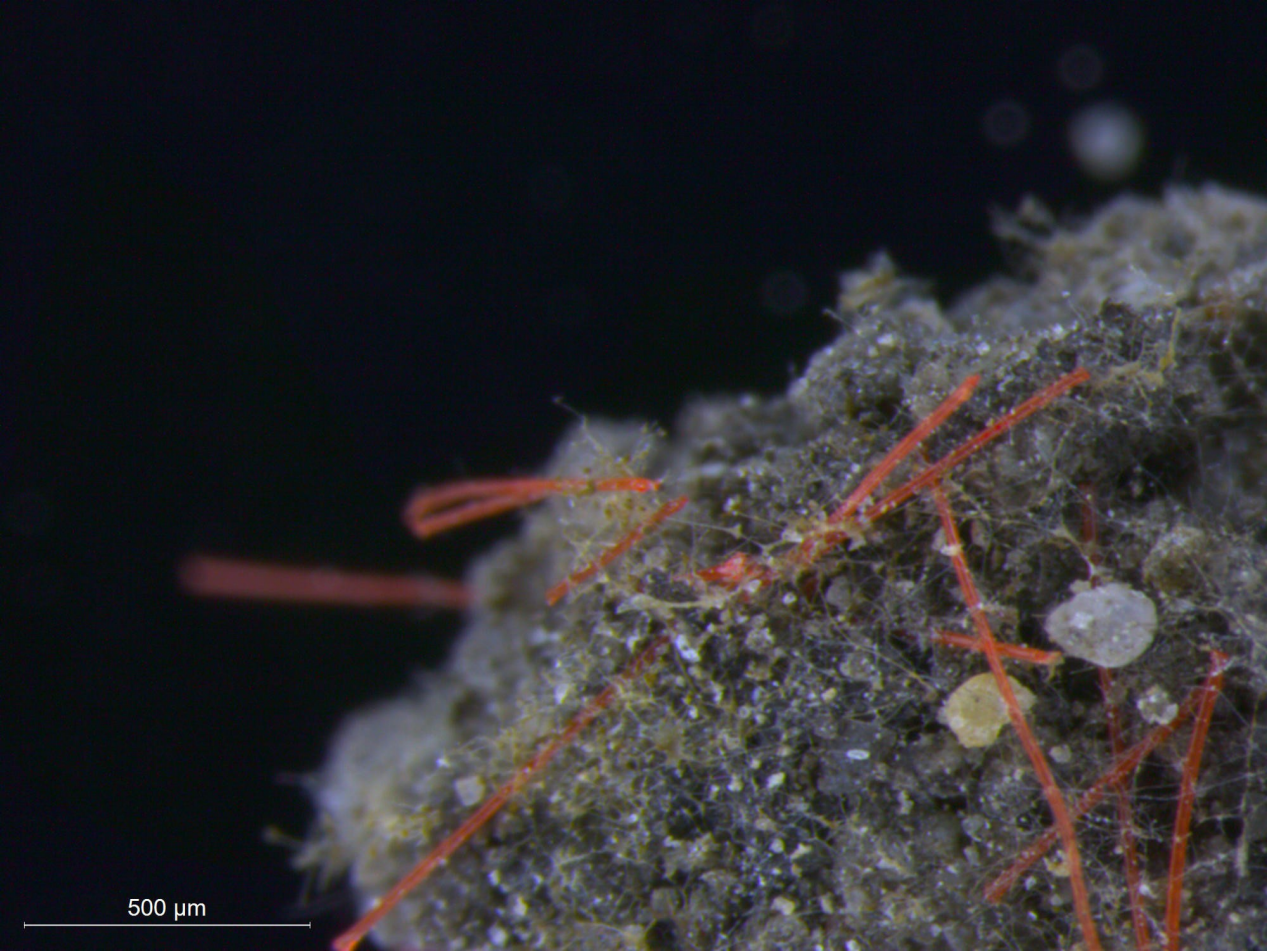
MP fibers, one of the many shapes in which the pollutant group MP appears in the environment, is shown interacting with a soil aggregate. The fibers (polypropylene) are orange, and the other filamentous objects are hyphae of soil fungi. Fibers have been frequently shown to affect soil aggregation in controlled experiments. The picture is taken from an experiment in which fibers were added. MP, microplastic. *Photo credit*: *Dr*. *Anika Lehmann (Freie Universität Berlin)*.

Plastic in the environment is an issue that was first reported from ocean observations several decades ago [1–3]. Research on microplastic (MP; plastic pieces smaller than 5 mm) also started in the ocean, perhaps because in aquatic systems these particles stand out more and can be noticed more easily, compared to a particle-rich environment such as soil [4]. Put a teaspoon of MP in a beaker of soil, stir, and it has all but “disappeared.” In addition, public attention was initially focused on oceans as a consequence of beach cleanups and the discovery of marine garbage patches. Clearly, MP is produced on land, either as primary or secondary MP, and thus is also expected to accumulate in soils of terrestrial ecosystems [5]. While we can learn much from results of aquatic MP work, it is also clear that effects in soils, despite many parallels, can be quite different from those in the aquatic environment: Most notably, MP appears to be interfering with the very fabric of the soil environment itself; by influencing soil bulk density and by affecting the stability of the building blocks of soil structure, the soil aggregates (Fig 1). In addition, there are unique research challenges in soil compared to aquatic ecosystems, a main impediment being harmonized extraction and quantification protocols.

Our knowledge of MP effects in soils is quite limited—considering that the first papers describing effects on soils appeared only a few years ago—but rapidly increasing [6,7]. Research has started with work taking a more ecotoxicological approach, focused on possible adverse effects on organisms [8]. By now, we think MP is probably best viewed as a factor of global change [9]. Why? A definition of a global change factor, as proposed in [10], stipulates the following: It is linked with human activity, it affects biota, and effects are apparent at a global scale. We believe MPs match these conditions. While this addition to the canon of factors of global change might take time to be fully appreciated [11], it is nevertheless important for MP research right now. This is the case for 3 reasons: (i) target concentrations of MP for experiments should not be just informed by current levels of contamination, but should be based on future, presumably higher levels. (ii) Proximate effects can be nominally positive, just as is the case for other factors of global change, such as elevated CO_2_ or warming. This does not mean that the effects are desirable; it merely means that increases in certain performance parameters (e.g., plant growth) can occur, but that these still represent deviations from the natural state. (iii) This realization of MP as a factor of global change shifts the research focus more toward ecosystems [12], with questions about feedbacks to the Earth system becoming more important and how MP might alter the functioning of terrestrial ecosystems, particularly the cycling of nutrients and carbon.

Carbon processing and storage in soils is a key ecosystem function, and we focus on this aspect here. We follow the path of carbon: starting with carbon entering the ecosystem, either as MP carbon or through net primary production, and then follow the fate of litter decomposition, and the interaction of MP with soil microbes, determining the fate of carbon in terms of storage or loss from the system (e.g., through respiration or leaching). We finish by briefly discussing nanoplastics (NPs) in a separate section, since effects are primarily expected to be toxicological in nature.

## Microplastic itself is also organic carbon

**Fig 2:**
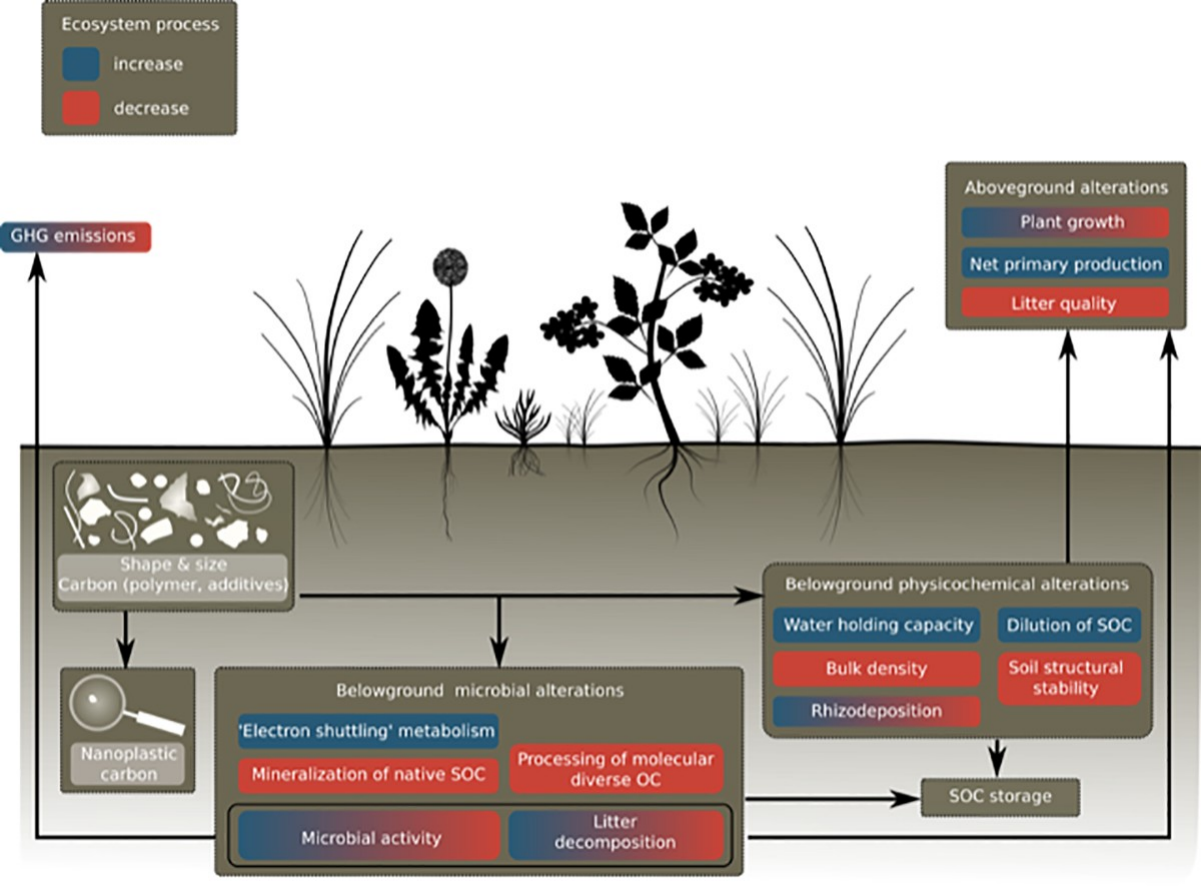
Potential effects of MP on soil organic carbon cycling in terrestrial ecosystems. Blue boxes indicate ecosystem process increases, and red boxes indicate decreases. Boxes with a color gradient from blue to red indicate that the process could be shifted in both directions. Arrows indicate hypothesized relationships between MP, SOC, and aboveground carbon cycling and GHG emissions. GHG, greenhouse gas; MP, microplastic; SOC, soil organic carbon.

The fact that MPs are particles that contain a lot of carbon, typically around 80% [13], makes them fairly unique in relation to other global change factors, potentially with the exception of pyrogenic carbon. MP carbon is thus already present in our soils, probably still making up only a tiny proportion of total soil organic matter carbon in most cases [13], but this could change in the future, and for specific ecosystems, such as urban and agricultural areas, since MP appears to be resistant to microbial decay compared to plant residues. A relatively small annual input compared to the large input by plants can translate to a quantitatively relevant accrual over long periods of time, as observed for pyrogenic carbon [14]. None of the current methods for assessing soil C are routinely able to distinguish soil organic matter carbon from this MP-C; this is troubling, since soil organic C storage is an ecosystem service, but even though MP-C is undeniably also organic carbon, it does not have the same origin and functionality as the rest of soil organic carbon, and it should not “count” in this context. We should separate these 2 organic carbon sources, but methods to do so routinely are not available, since MP quantification protocols are still being developed and refined. MP particles, once they arrive at the soil surface, can quickly become incorporated into the soil matrix [15–17]; MP forms its own cycle [18], main features of which are slow MP decomposition, and potential loss to other environmental compartments, e.g., via leaching to groundwater or via erosion to lower slope positions. The following sections, covering different spatiotemporal scales from large to small, are about the effects the accumulating MP may have in soil (Fig 2).

## Plant growth and net primary production

MP can affect plant growth through a variety of different mechanisms [19], which are thought to be indirect via the effect MP has on soil and soil biota. Examples of such indirect effects are changes in soil structure and bulk density, which can affect root penetration resistance, changes in water holding capacity, and others. Biodegradable plastics might induce nutrient immobilization as these carbon-rich particles are being decomposed by the soil microbial community. In addition, key plant symbionts, such as root-colonizing mycorrhizal fungi, might be affected by MPs or their effects on soil physicochemical properties. In some cases, resulting effects of MP on plant growth have been positive [20,21], but there are also reports of negative effects [22]. Such differences are explained by the fact that different MPs (including their chemical additives, some of which might be toxic), soils, and plants were used in these different studies, but it is not clear how each of these factors contributed to observed effects. Nor is it clear whether even major mechanisms by which MP may affect plant growth are understood. MP likely has different effects on different plant species in a plant community, which would explain the shift in grassland plant community composition that was observed following addition of MP fibers to soil [23].

## Microplastic affecting litter decomposition

There are 2 broad mechanisms through which MP could affect a major ecosystem process, litter decomposition: (i) MP could induce shifts in litter quality; and (ii) MP could affect the decomposition process itself. We discuss these 2 mechanisms in the following.

### Shifts in litter quality

Improved plant performance through MP addition can lead to increased above- and belowground production of biomass, which will become litter during senescence. Assuming that MP increases plant growth unrelated to nutrient availability in the soil, foliar nutrient contents could decrease (“dilution effect”), resulting in reduced decomposition rates. Specifically, the C:N ratio of plant litter is known to affect its decomposition rate [24]. However, a slower decomposition of leaf litter, caused by reduced microbial activity, might also reduce carbon stabilization in soil organic matter, as microbial products of decomposition are the main component of soil aggregation and the formation of organo–mineral interactions with the mineral soil matrix [25]. During the decay of litter with lower quality, microbes might mine for nitrogen in the soil organic matter in order to process the C from the litter, thus inducing a C loss from the soil [26].

Dilution of litter available to decomposers through the presence of MP may reduce decomposition, similar to observations made with additions of other slow-mineralizing C additions. Changes in nutrient or water availability as well as soil structure and aggregation may also affect the plant community in natural ecosystems or possibly weed composition in agricultural systems. Such changes in plant composition will also alter the composition of the litter and its decomposition.

Another potential way MP could influence plant litter quality is via effects on the microbial community, including arbuscular mycorrhizal fungi, which play a key role in nutrient mobilization for plants and also in interactions with decomposers [27,28]. In some cases, root colonization by arbuscular mycorrhiza was increased by some MP types [20], but how this affects plant nutrient contents or the decomposer community has not been addressed yet.

### Affecting the decomposition process

MP changes important physicochemical soil properties that are important environmental parameters for soil biota: the size of soil aggregates (mean weight diameter), soil porosity (aeration), and water holding capacity. Changes in microbial community composition have been observed in several cases and can have cascading effects on litter decomposition. Changes in microbial activity (e.g., [20]) can be linked to (i) altered physicochemical soil properties; (ii) direct toxic effects of MP, its additives, or sorbed contaminants (in case of negative effects); and (iii) supply with microbially available organic carbon and nitrogen for some plastic types. The carbon in MP can cause a “priming” effect, i.e., increased microbial activity with potential changes in nutrient availability and dissolved organic C, but the large C:N ratio of most types of MP can also induce the immobilization of nutrients, and thus decrease microbial activity. Specific MP types, such as tire wear particles, are able to change the soil pH with consequences for the availability of nutrients and heavy metals [29]. There are direct effects of the presence of MP on soil biota that play a crucial role in decomposition. Key organisms for the incorporation of litter into the soil are earthworms (Lumbricidae). MP can cause skin lesions, increase mortality, and reduce reproductive rates in earthworms [30], thus reducing the transport of organic matter into deeper soil layers. Earthworms are a larger group of the soil biota with actual intake of MP particles and potentially fragmenting MP particles during digestion [31]. Upon excretion, these particles become available to other soil organisms of the food web, e.g., smaller decomposers, such as microarthropods [32].

## Rhizodeposition

Rhizodeposition is a major pathway of organic C input into soils [33], creating a hotspot of microbial activity, the rhizosphere (the zone under immediate influence by roots, enriched in organic C). Rhizodeposition is influenced by a very wide range of environmental factors [34], and it is almost certain from first principles that it will be affected by MP in soils. However, it is not clear how rhizodeposition would be precisely affected. Shifts in rhizodeposited organic C quality or quantity would have far-reaching effects on microbial communities and their functions, specifically organic C stabilization, as belowground C input is now thought to be more effective in sequestering organic C than leaf litter [35].

## Potential mechanisms of direct MP interaction with microbes in the context of soil organic C cycling

Very little is known about how MP particles in soil would interact with microbes on their surfaces; in aquatic environments, the microbially colonized plastic surface has been termed the “ecocorona” [36], and what this means for organic C cycling. In the following, we present several testable mechanistic hypotheses for this interaction; some of these ideas are borrowed from the study of pyrogenic C particles.

### Electrochemistry “electron shuttling” hypothesis

Even though plastic tends to generally be a poor conductor, MP particles, especially after weathering, could have surface functional groups (e.g., ketones) that are redox active, especially after weathering in soil, and thus attract microbes who use them as electron sinks or donors in their metabolism. If this process works similarly to biochar—that is, if the electron donation/accepting is faster and potentially greater than for other organic matter, which makes microbial metabolism more energy efficient [37]—organic C transformation would change. This may result in faster decomposition [38] and lower methane emissions [14].

### “Microbial frustration” hypothesis

Functional complexity has recently been hypothesized to increase the persistence of soil organic carbon, and one aspect of this functional complexity is molecular diversity of C compounds [39]. MP could increase the molecular diversity of soil C, by adding novel polymers, and especially through the broad range of additives they contain. This might make it harder for microbes overall to process organic C.

### Negative or positive priming hypothesis

Priming is a mechanism that describes how the addition of a carbon substrate affects the mineralization of native soil organic C; there can be positive priming, if the substrate addition favors the mineralization of existing soil organic C, or negative priming, if the opposite is true. In parallel to work on pyrogenic C [40], negative priming could be due to dilution, adsorption of soil dissolved organic C to plastic surfaces (organo–organo persistence hypothesis), or initially due to substrate switching by which easily mineralizable organic C in MP is preferentially metabolized. Positive priming could be due to co-metabolism; this effect is likely minimal given the persistence of MP particles, but could be caused by easily metabolizable additives or could play a role with biodegradable plastics.

## Nanoplastics

In this paper, we focused on MP. However, if MP particles undergo further fragmentation, they can form even smaller particles, eventually potentially reaching nanoparticle size (<100 nm), which can differ in behavior and effect from MP. For example, it is possible that NP particles are taken up into plants [41], a finding obtained using hydroponic growth conditions that may not be directly transferable to soils [42]. NP particles cannot be readily quantified in soils at the moment, but they would still also represent carbon. It is likely that effects will shift to direct chemical toxicity, compared to the more indirect effects of MP, as already shown for plants [43]. Analytical methods for NP are currently under development, and it appears that NP particles are indeed present in the soil matrix [44]. Consequences of NP for soils are unclear since there are only very few studies [45,46], and thus it is presently not understood how they would influence ecosystem processes, such as soil C processing and storage. Several of the chemical effects of MP should apply to NP, however, with biological effects exacerbated by the fact that NPs can be taken up by plants and possibly microbes, whereas physical effects on soil structure may be less important.

## Conclusions

MPs have potentially pervasive influences on terrestrial ecosystems, as exemplified here for the processes related to soil carbon storage. Examining these effects in detail will be an immense challenge, because MP particles come in a dizzying diversity of types [47] (including size, shape [48], aging-related modification [49], and nature of additives [50,51]) that very likely differ quite drastically in their effects. It is not clear how this need to account for the diversity of MP types can be reconciled with the logistic demands on ecosystem-level experimentation, such as in mesocosm or field experiments. Also, mechanisms of interaction of MP with soil biota will be very sensitive to these different properties. However, some initial ideas about how to bring order into this complex parameter space are emerging, for example, for particle shape [48]; such hypotheses are also needed for other key aspects, so that representative and environmentally relevant MP types (or mixtures) can be selected for use in larger scale experiments.
